# *catena*-Poly[[tetra­kis­(hexa­methyl­phospho­ramide-κ*O*)bis­(nitrato-κ^2^ *O*,*O*′)yttrium(III)] [silver(I)-di-μ-sulfido-molybdenum(VI)-di-μ-sulfido]]

**DOI:** 10.1107/S1600536811030996

**Published:** 2011-08-06

**Authors:** Jinfang Zhang

**Affiliations:** a Molecular Materials Research Center, Scientific Research Academy, School of Chemistry and Chemical Engineering, Jiangsu University, Zhenjiang 212013, People’s Republic of China

## Abstract

In the cation of the title compound, {[Y(NO_3_)_2_(C_6_H_18_N_3_OP)_4_][AgMoS_4_]}_*n*_, the Y atom is coordinated by eight O atoms from two chelating nitrate groups and four hexa­methyl­phospho­ramide (hmp) ligands, which gives rise to a distorted square-anti­prismatic environment. Together with the two nitrate ligands, the overall charge for the complex cation is +1, which leads to the anionic chain having a monovalent repeat unit. The polymeric anionic chain, with Mo—Ag—Mo and Ag—Mo—Ag angles of 161.916 (13) and 153.915 (13)°, respectively, presents a distorted linear configuration. The cations and the anions are linked *via* weak C—H⋯S hydrogen-bonding inter­actions while the cations exhibit inter­molecular C—H⋯O inter­actions. The structure is isotypic with the corresponding W, Yb, Eu, Nd, La and Dy complexes.

## Related literature

For one-dimensional Mo(W)/S/Ag anionic polymers, see: Niu *et al.* (2004 ▶) and for their properties, see: Zhang, Song *et al.* (2007 ▶). For isotypic W, Yb, Eu, Nd, La and Dy complexes, see: Zhang, Cao *et al.* (2007 ▶); Cao *et al.* (2007 ▶); Zhang, Qian *et al.* (2007 ▶); Tang, Zhang & Zhang (2008 ▶); Tang, Zhang, Zhang & Lu (2008 ▶); Zhang (2010 ▶), respectively.
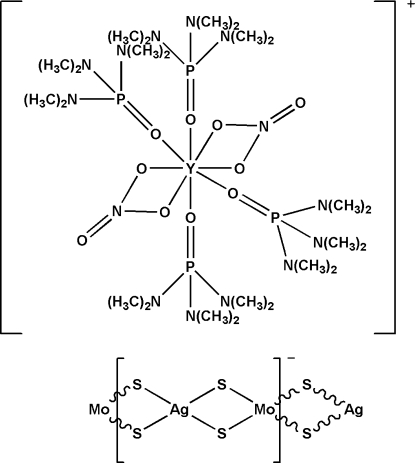


## Experimental

### 

#### Crystal data

[Y(NO_3_)_2_(C_6_H_18_N_3_OP)_4_][AgMoS_4_]
*M* *_r_* = 1261.84
Monoclinic, 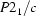

*a* = 15.777 (3) Å
*b* = 29.650 (6) Å
*c* = 11.339 (2) Å
β = 90.90 (3)°
*V* = 5303.6 (17) Å^3^
*Z* = 4
Mo *K*α radiation
μ = 2.02 mm^−1^
*T* = 153 K
0.35 × 0.20 × 0.15 mm

#### Data collection

Rigaku Saturn724+ diffractometer
Absorption correction: multi-scan (*CrystalClear*; Rigaku, 2007 ▶) *T*_min_ = 0.622, *T*_max_ = 0.739
25568 measured reflections
10435 independent reflections
8978 reflections with *I* > 2σ(*I*)
*R*_int_ = 0.033

#### Refinement

*R*[*F* ^2^ > 2σ(*F* ^2^)] = 0.045
*wR*(*F* ^2^) = 0.091
*S* = 1.02
10435 reflections
556 parameters
H-atom parameters constrained
Δρ_max_ = 0.86 e Å^−3^
Δρ_min_ = −0.79 e Å^−3^

### 

Data collection: *CrystalClear* (Rigaku, 2007 ▶); cell refinement: *CrystalClear*; data reduction: *CrystalClear*; program(s) used to solve structure: *SHELXS97* (Sheldrick, 2008 ▶); program(s) used to refine structure: *SHELXL97* (Sheldrick, 2008 ▶); molecular graphics: *SHELXTL* (Sheldrick, 2008 ▶); software used to prepare material for publication: *SHELXTL*.

## Supplementary Material

Crystal structure: contains datablock(s) I, global. DOI: 10.1107/S1600536811030996/pv2435sup1.cif

Structure factors: contains datablock(s) I. DOI: 10.1107/S1600536811030996/pv2435Isup2.hkl

Additional supplementary materials:  crystallographic information; 3D view; checkCIF report

## Figures and Tables

**Table 1:** Hydrogen-bond geometry (Å, °)

*D*—H⋯*A*	*D*—H	H⋯*A*	*D*⋯*A*	*D*—H⋯*A*
C5—H5*A*⋯S2^i^	0.96	2.79	3.710 (6)	160
C16—H16*A*⋯O10^ii^	0.96	2.49	3.292 (6)	141
C18—H18*A*⋯O10^iii^	0.96	2.54	3.470 (9)	162

Symmetry codes: (i) 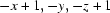; (ii) 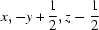; (iii) 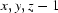.

**Fig. 1.**
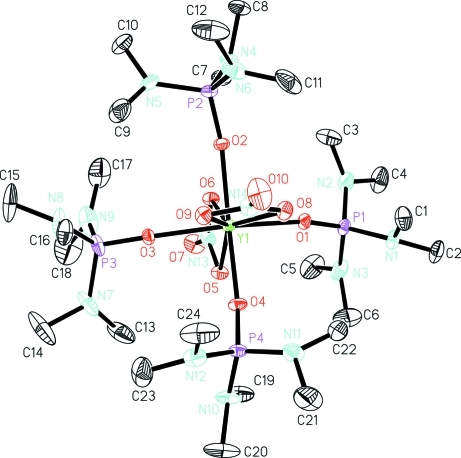
The molecular structure of the cation in the title compound, with atom labels and 30% probability displacement ellipsoids. All H atoms have been omitted.

**Fig. 2.**
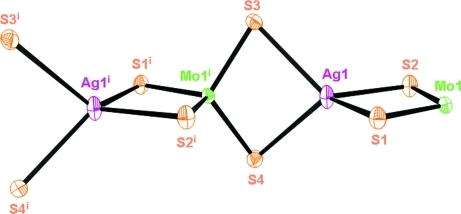
The molecular structure of a portion of the anionic chain in the title compound, with atom labels and 30% probability displacement ellipsoids, Symmetry code: (i) x, 1/2 - y, -1/2 + z.

## supplementary crystallographic information

### Comment

One-dimensional Mo(W)/S/Ag anionic polymers have attracted much attention for their configurational isomerism (Niu *et al.*, 2004) and unique properties as functional materials, such as third-order nonlinear optical (NLO) materials (Zhang, Song *et al.*, 2007). Different solvent-coordinated rare-earth cations proved effective to obtain various configurations of anionic chains (Niu *et al.*, 2004). In hexamethylphosphoramide (hmp), tetrathiomolybdate, silver iodide and dysprosium nitrate were self-assembled to form a one-dimensional anionic [AgMoS_4_]^-^ chain in the compound, {[bi(nitrato-*κ^2^O,O'*)tetrakis(hexamethylphosphoramide-*κO*) yttrium(III)]*catena*-[tetra-*µ*_2_-sulfidosilver(I)molybdenum(VI)\ ]} with a formula of {[Y(hmp)_4_(NO_3_)_2_][MoS_4_Ag]}_n_.

In the title complex, Y^3-^ in the cation is coordinated by eight O atoms from two nitrate and four hmp ligands. In the presence of two nitrate ligands, the cation in the title compound is univalent (Fig. 1). The anionic chain in the title compound (Fig. 2) has a distorted linear configuration with Mo—Ag—Mo and Ag—Mo—Ag angles 161.916 (13) and 153.915 (13) °, respectively. The cations and the anions are linked *via* weak hydrogen bondeing interactions of the type C—H···S while the cations exhibit C—H···O type intermolecular interactions (Table 1). The title complex is isostructural with W (Zhang, Cao *et al.*, 2007), Yb (Cao *et al.*, 2007), Eu (Zhang, Qian *et al.*, 2007), Nd (Tang, Zhang & Zhang, 2008), La (Tang, Zhang, Zhang & Lu, 2008) and Dy (Zhang, 2010) isomorphs.

### Experimental

AgI (2 mmol) was added to a solution of [NH_4_]_2_MoS_4_ (2 mmol, in 28 mL hmp) with thorough stirring for 1.5 h. The solution was stirred for two minute after Y(NO_3_)_3_.6H_2_O (1 mmol) was added. After filtration the black-red filtrate was carefully laid on the surface with 30 ml *i*-PrOH. Black-red block crystals were obtained after about two weeks.

### Refinement

H atoms were positioned geometrically and refined with riding model, with *U*_iso_ = 1.5*U*_eq_ for methyl H atoms and 0.96 Å for C—H bonds.

### Crystal data

**Table:**

[Y(NO_3_)_2_(C_6_H_18_N_3_OP)_4_][AgMoS_4_]	*F*(000) = 2584
*M**_r_* = 1261.84	*D*_x_ = 1.580 Mg m^−^^3^
Monoclinic, *P*2_1_/*c*	Mo *K*α radiation, λ = 0.71073 Å
Hall symbol: -P 2ybc	Cell parameters from 22373 reflections
*a* = 15.777 (3) Å	θ = 3.0–29.1°
*b* = 29.650 (6) Å	µ = 2.02 mm^−^^1^
*c* = 11.339 (2) Å	*T* = 153 K
β = 90.90 (3)°	Block, black-red
*V* = 5303.6 (17) Å^3^	0.35 × 0.20 × 0.15 mm
*Z* = 4	

### Data collection

**Table:**

Rigaku Saturn724+ diffractometer	10435 independent reflections
Radiation source: fine-focus sealed tube	8978 reflections with *I* > 2σ(*I*)
graphite	*R*_int_ = 0.033
dtprofit.ref scans	θ_max_ = 26.0°, θ_min_ = 3.0°
Absorption correction: multi-scan (*CrystalClear*; Rigaku, 2007)	*h* = −19→16
*T*_min_ = 0.622, *T*_max_ = 0.739	*k* = −31→36
25568 measured reflections	*l* = −12→13

### Refinement

**Table:**

Refinement on *F*^2^	Primary atom site location: structure-invariant direct methods
Least-squares matrix: full	Secondary atom site location: difference Fourier map
*R*[*F*^2^ > 2σ(*F*^2^)] = 0.045	Hydrogen site location: inferred from neighbouring sites
*wR*(*F*^2^) = 0.091	H-atom parameters constrained
*S* = 1.02	*w* = 1/[σ^2^(*F*_o_^2^) + (0.0325*P*)^2^ + 8.8309*P*] where *P* = (*F*_o_^2^ + 2*F*_c_^2^)/3
10435 reflections	(Δ/σ)_max_ = 0.001
556 parameters	Δρ_max_ = 0.86 e Å^−^^3^
0 restraints	Δρ_min_ = −0.79 e Å^−^^3^

### Special details

**Table:**

Geometry. All e.s.d.'s (except the e.s.d. in the dihedral angle between two l.s. planes) are estimated using the full covariance matrix. The cell e.s.d.'s are taken into account individually in the estimation of e.s.d.'s in distances, angles and torsion angles; correlations between e.s.d.'s in cell parameters are only used when they are defined by crystal symmetry. An approximate (isotropic) treatment of cell e.s.d.'s is used for estimating e.s.d.'s involving l.s. planes.
Refinement. Refinement of *F*^2^ against ALL reflections. The weighted *R*-factor *wR* and goodness of fit *S* are based on *F*^2^, conventional *R*-factors *R* are based on *F*, with *F* set to zero for negative *F*^2^. The threshold expression of *F*^2^ > σ(*F*^2^) is used only for calculating *R*-factors(gt) *etc*. and is not relevant to the choice of reflections for refinement. *R*-factors based on *F*^2^ are statistically about twice as large as those based on *F*, and *R*- factors based on ALL data will be even larger.

### Fractional atomic coordinates and isotropic or equivalent isotropic displacement parameters (Å^2^)

**Table:**

	*x*	*y*	*z*	*U*_iso_*/*U*_eq_	
Y1	0.76202 (2)	0.082657 (11)	0.67216 (3)	0.02106 (9)	
P1	0.80109 (7)	−0.03027 (3)	0.80037 (9)	0.0302 (2)	
P2	0.97799 (6)	0.13259 (4)	0.67874 (9)	0.0279 (2)	
P3	0.70659 (8)	0.14681 (4)	0.40283 (10)	0.0411 (3)	
P4	0.54196 (6)	0.09637 (3)	0.76889 (10)	0.0289 (2)	
O1	0.79306 (16)	0.01827 (8)	0.7709 (2)	0.0286 (6)	
O2	0.89658 (15)	0.10701 (9)	0.6769 (2)	0.0280 (6)	
O3	0.72762 (16)	0.12679 (8)	0.5191 (2)	0.0303 (6)	
O4	0.62457 (16)	0.08067 (8)	0.7201 (2)	0.0311 (6)	
O5	0.69836 (16)	0.02710 (9)	0.5347 (3)	0.0327 (6)	
O6	0.83115 (16)	0.04107 (9)	0.5110 (2)	0.0316 (6)	
O7	0.7627 (2)	0.00151 (11)	0.3795 (3)	0.0509 (8)	
O8	0.77629 (17)	0.10293 (8)	0.8803 (2)	0.0329 (6)	
O9	0.74838 (17)	0.15805 (9)	0.7615 (2)	0.0328 (6)	
O10	0.7740 (3)	0.17164 (11)	0.9480 (3)	0.0663 (11)	
N1	0.7789 (2)	−0.03678 (11)	0.9404 (3)	0.0371 (8)	
N2	0.8939 (2)	−0.04824 (13)	0.7609 (4)	0.0507 (11)	
N3	0.7348 (3)	−0.06527 (12)	0.7321 (3)	0.0458 (10)	
N4	1.05558 (19)	0.09750 (12)	0.6519 (3)	0.0322 (8)	
N5	0.9777 (2)	0.17290 (12)	0.5800 (4)	0.0444 (10)	
N6	0.9940 (2)	0.15647 (15)	0.8057 (3)	0.0511 (11)	
N7	0.6047 (3)	0.13896 (15)	0.3742 (4)	0.0727 (15)	
N8	0.7272 (3)	0.20021 (12)	0.4047 (3)	0.0525 (11)	
N9	0.7609 (4)	0.12405 (15)	0.2997 (4)	0.0691 (14)	
N10	0.4666 (2)	0.07045 (13)	0.6975 (3)	0.0422 (9)	
N11	0.5444 (2)	0.08704 (14)	0.9118 (3)	0.0465 (10)	
N12	0.5170 (2)	0.14923 (12)	0.7602 (4)	0.0480 (10)	
N13	0.7639 (2)	0.02248 (11)	0.4719 (3)	0.0317 (8)	
N14	0.7659 (2)	0.14511 (11)	0.8656 (3)	0.0358 (8)	
C1	0.8019 (3)	−0.00241 (16)	1.0252 (4)	0.0525 (13)	
H1A	0.7606	−0.0015	1.0863	0.079*	
H1B	0.8040	0.0264	0.9866	0.079*	
H1C	0.8566	−0.0092	1.0591	0.079*	
C2	0.7730 (3)	−0.08223 (15)	0.9925 (4)	0.0526 (13)	
H2A	0.8274	−0.0909	1.0241	0.079*	
H2B	0.7554	−0.1034	0.9329	0.079*	
H2C	0.7323	−0.0820	1.0546	0.079*	
C3	0.9646 (3)	−0.01683 (18)	0.7541 (5)	0.0639 (16)	
H3A	0.9934	−0.0154	0.8292	0.096*	
H3B	0.9436	0.0126	0.7334	0.096*	
H3C	1.0033	−0.0269	0.6952	0.096*	
C4	0.9180 (4)	−0.09588 (19)	0.7776 (5)	0.0766 (19)	
H4A	0.9565	−0.1047	0.7170	0.115*	
H4B	0.8682	−0.1144	0.7733	0.115*	
H4C	0.9451	−0.0995	0.8534	0.115*	
C5	0.7462 (4)	−0.08073 (19)	0.6131 (5)	0.0776 (19)	
H5A	0.7366	−0.1127	0.6094	0.116*	
H5B	0.8030	−0.0742	0.5891	0.116*	
H5C	0.7067	−0.0656	0.5615	0.116*	
C6	0.6461 (4)	−0.0679 (2)	0.7649 (6)	0.084 (2)	
H6A	0.6119	−0.0522	0.7075	0.126*	
H6B	0.6390	−0.0543	0.8410	0.126*	
H6C	0.6289	−0.0989	0.7678	0.126*	
C7	1.0464 (3)	0.06450 (15)	0.5568 (4)	0.0413 (11)	
H7A	1.0676	0.0771	0.4851	0.062*	
H7B	0.9876	0.0569	0.5462	0.062*	
H7C	1.0780	0.0378	0.5767	0.062*	
C8	1.1434 (3)	0.10681 (18)	0.6883 (4)	0.0482 (12)	
H8A	1.1699	0.0794	0.7144	0.072*	
H8B	1.1439	0.1283	0.7516	0.072*	
H8C	1.1738	0.1188	0.6227	0.072*	
C9	0.9107 (4)	0.20743 (17)	0.5839 (6)	0.0700 (17)	
H9A	0.9329	0.2343	0.6199	0.105*	
H9B	0.8642	0.1964	0.6291	0.105*	
H9C	0.8914	0.2141	0.5051	0.105*	
C10	1.0469 (3)	0.18315 (17)	0.5010 (5)	0.0571 (14)	
H10A	1.0245	0.1893	0.4235	0.086*	
H10B	1.0847	0.1578	0.4977	0.086*	
H10C	1.0772	0.2091	0.5297	0.086*	
C11	0.9814 (4)	0.1307 (3)	0.9123 (5)	0.086 (2)	
H11A	1.0349	0.1263	0.9522	0.129*	
H11B	0.9571	0.1019	0.8925	0.129*	
H11C	0.9437	0.1468	0.9630	0.129*	
C12	1.0358 (4)	0.2006 (2)	0.8221 (6)	0.086 (2)	
H12A	0.9981	0.2208	0.8617	0.129*	
H12B	1.0499	0.2129	0.7466	0.129*	
H12C	1.0866	0.1968	0.8688	0.129*	
C13	0.5659 (4)	0.0960 (2)	0.3871 (6)	0.086 (2)	
H13A	0.5116	0.0996	0.4232	0.129*	
H13B	0.6013	0.0771	0.4358	0.129*	
H13C	0.5584	0.0823	0.3109	0.129*	
C14	0.5508 (5)	0.1719 (2)	0.3134 (7)	0.118 (3)	
H14A	0.5523	0.1667	0.2299	0.177*	
H14B	0.5711	0.2018	0.3302	0.177*	
H14C	0.4936	0.1690	0.3401	0.177*	
C15	0.7525 (5)	0.22617 (18)	0.3010 (5)	0.091 (2)	
H15A	0.7048	0.2429	0.2710	0.137*	
H15B	0.7722	0.2059	0.2412	0.137*	
H15C	0.7973	0.2466	0.3228	0.137*	
C16	0.6976 (3)	0.22754 (14)	0.5052 (4)	0.0491 (13)	
H16A	0.7369	0.2517	0.5201	0.074*	
H16B	0.6940	0.2088	0.5740	0.074*	
H16C	0.6427	0.2398	0.4866	0.074*	
C17	0.8509 (5)	0.1213 (2)	0.3117 (6)	0.087 (2)	
H17A	0.8689	0.0909	0.2983	0.131*	
H17B	0.8678	0.1305	0.3898	0.131*	
H17C	0.8767	0.1409	0.2550	0.131*	
C18	0.7224 (7)	0.1064 (3)	0.1906 (6)	0.132 (4)	
H18A	0.7484	0.1205	0.1240	0.197*	
H18B	0.6628	0.1129	0.1894	0.197*	
H18C	0.7308	0.0744	0.1869	0.197*	
C19	0.4800 (3)	0.02788 (17)	0.6395 (5)	0.0615 (16)	
H19A	0.4635	0.0038	0.6908	0.092*	
H19B	0.5389	0.0248	0.6211	0.092*	
H19C	0.4466	0.0267	0.5681	0.092*	
C20	0.3765 (3)	0.0808 (2)	0.7169 (6)	0.078 (2)	
H20A	0.3465	0.0810	0.6426	0.116*	
H20B	0.3718	0.1099	0.7536	0.116*	
H20C	0.3526	0.0583	0.7672	0.116*	
C21	0.4702 (4)	0.0970 (3)	0.9843 (5)	0.083 (2)	
H21A	0.4292	0.0733	0.9751	0.124*	
H21B	0.4455	0.1251	0.9595	0.124*	
H21C	0.4876	0.0991	1.0657	0.124*	
C22	0.5925 (3)	0.04816 (19)	0.9571 (5)	0.0579 (14)	
H22A	0.6044	0.0523	1.0397	0.087*	
H22B	0.6448	0.0456	0.9155	0.087*	
H22C	0.5598	0.0212	0.9458	0.087*	
C23	0.4854 (4)	0.1689 (2)	0.6514 (6)	0.084 (2)	
H23A	0.4446	0.1919	0.6689	0.126*	
H23B	0.4590	0.1459	0.6039	0.126*	
H23C	0.5316	0.1820	0.6093	0.126*	
C24	0.5484 (3)	0.18326 (18)	0.8443 (7)	0.088 (2)	
H24A	0.5892	0.2022	0.8064	0.132*	
H24B	0.5746	0.1685	0.9108	0.132*	
H24C	0.5019	0.2014	0.8705	0.132*	
Ag1	0.28258 (2)	0.234941 (11)	0.28632 (3)	0.03952 (10)	
Mo1	0.28386 (2)	0.272038 (10)	0.52560 (3)	0.02233 (9)	
S1	0.28577 (7)	0.31537 (3)	0.36778 (9)	0.0317 (2)	
S2	0.28360 (7)	0.19915 (3)	0.48461 (9)	0.0352 (2)	
S3	0.39752 (7)	0.21324 (4)	0.13208 (9)	0.0378 (3)	
S4	0.16939 (7)	0.21227 (4)	0.12641 (9)	0.0364 (3)	

### Atomic displacement parameters (Å^2^)

**Table:**

	*U*^11^	*U*^22^	*U*^33^	*U*^12^	*U*^13^	*U*^23^
Y1	0.01862 (18)	0.01585 (18)	0.0287 (2)	0.00010 (14)	0.00161 (15)	0.00090 (14)
P1	0.0377 (6)	0.0205 (5)	0.0326 (6)	0.0066 (5)	0.0093 (5)	0.0066 (4)
P2	0.0209 (5)	0.0341 (6)	0.0288 (5)	−0.0052 (4)	0.0006 (4)	−0.0057 (4)
P3	0.0625 (8)	0.0261 (6)	0.0340 (6)	0.0101 (6)	−0.0188 (6)	−0.0007 (5)
P4	0.0199 (5)	0.0265 (5)	0.0406 (6)	0.0010 (4)	0.0037 (4)	−0.0072 (5)
O1	0.0334 (15)	0.0177 (13)	0.0347 (15)	0.0030 (11)	0.0023 (12)	0.0047 (11)
O2	0.0202 (13)	0.0299 (14)	0.0341 (15)	−0.0032 (11)	0.0017 (11)	0.0005 (12)
O3	0.0327 (15)	0.0236 (14)	0.0343 (16)	0.0016 (12)	−0.0055 (12)	0.0072 (12)
O4	0.0202 (13)	0.0273 (14)	0.0461 (17)	0.0004 (11)	0.0070 (12)	−0.0061 (12)
O5	0.0262 (15)	0.0275 (14)	0.0442 (17)	0.0004 (12)	−0.0006 (13)	−0.0062 (13)
O6	0.0261 (14)	0.0297 (15)	0.0389 (16)	−0.0016 (12)	0.0001 (12)	−0.0057 (13)
O7	0.055 (2)	0.057 (2)	0.0414 (19)	0.0048 (17)	−0.0023 (16)	−0.0232 (17)
O8	0.0421 (17)	0.0208 (14)	0.0358 (16)	0.0028 (12)	0.0050 (13)	−0.0014 (12)
O9	0.0387 (16)	0.0217 (14)	0.0379 (17)	0.0019 (12)	−0.0008 (13)	−0.0035 (12)
O10	0.108 (3)	0.0392 (19)	0.051 (2)	0.014 (2)	−0.012 (2)	−0.0215 (17)
N1	0.053 (2)	0.0271 (18)	0.0318 (19)	0.0061 (17)	0.0107 (17)	0.0075 (15)
N2	0.046 (2)	0.042 (2)	0.065 (3)	0.0185 (19)	0.017 (2)	0.021 (2)
N3	0.069 (3)	0.0247 (18)	0.044 (2)	−0.0089 (19)	0.013 (2)	−0.0039 (17)
N4	0.0211 (16)	0.042 (2)	0.0333 (19)	−0.0021 (15)	0.0008 (14)	−0.0064 (16)
N5	0.034 (2)	0.035 (2)	0.065 (3)	−0.0059 (17)	0.0132 (19)	0.0097 (19)
N6	0.037 (2)	0.077 (3)	0.039 (2)	0.001 (2)	−0.0042 (18)	−0.025 (2)
N7	0.074 (3)	0.049 (3)	0.093 (4)	0.016 (2)	−0.056 (3)	−0.001 (2)
N8	0.100 (3)	0.0285 (19)	0.029 (2)	0.010 (2)	0.004 (2)	0.0087 (16)
N9	0.113 (4)	0.051 (3)	0.043 (3)	0.029 (3)	−0.005 (3)	−0.010 (2)
N10	0.0196 (17)	0.053 (2)	0.054 (2)	−0.0008 (16)	0.0040 (16)	−0.0263 (19)
N11	0.036 (2)	0.064 (3)	0.040 (2)	0.0086 (19)	0.0055 (17)	−0.0086 (19)
N12	0.037 (2)	0.031 (2)	0.076 (3)	0.0032 (17)	0.010 (2)	−0.008 (2)
N13	0.035 (2)	0.0238 (17)	0.036 (2)	0.0065 (15)	−0.0016 (16)	−0.0043 (15)
N14	0.038 (2)	0.030 (2)	0.039 (2)	0.0040 (16)	0.0026 (17)	−0.0066 (17)
C1	0.074 (4)	0.050 (3)	0.034 (3)	0.005 (3)	−0.005 (2)	−0.001 (2)
C2	0.068 (3)	0.042 (3)	0.048 (3)	0.010 (2)	0.021 (3)	0.019 (2)
C3	0.036 (3)	0.066 (4)	0.089 (4)	0.004 (3)	−0.011 (3)	−0.017 (3)
C4	0.088 (4)	0.060 (4)	0.082 (4)	0.041 (3)	0.035 (4)	0.029 (3)
C5	0.105 (5)	0.058 (4)	0.070 (4)	−0.026 (3)	0.013 (4)	−0.024 (3)
C6	0.059 (4)	0.091 (5)	0.102 (5)	−0.023 (3)	0.008 (4)	−0.023 (4)
C7	0.032 (2)	0.049 (3)	0.044 (3)	0.001 (2)	0.003 (2)	−0.012 (2)
C8	0.024 (2)	0.071 (3)	0.049 (3)	0.002 (2)	−0.001 (2)	−0.009 (3)
C9	0.063 (4)	0.042 (3)	0.106 (5)	0.007 (3)	0.020 (3)	0.019 (3)
C10	0.055 (3)	0.050 (3)	0.067 (4)	−0.013 (3)	0.019 (3)	0.010 (3)
C11	0.052 (3)	0.172 (7)	0.032 (3)	0.019 (4)	−0.006 (3)	−0.003 (4)
C12	0.060 (4)	0.094 (5)	0.104 (5)	−0.014 (3)	−0.013 (4)	−0.064 (4)
C13	0.059 (4)	0.073 (4)	0.125 (6)	0.006 (3)	−0.046 (4)	−0.017 (4)
C14	0.127 (6)	0.088 (5)	0.136 (7)	0.049 (5)	−0.085 (6)	−0.008 (5)
C15	0.181 (8)	0.048 (3)	0.046 (3)	0.030 (4)	0.028 (4)	0.024 (3)
C16	0.080 (4)	0.028 (2)	0.039 (3)	0.004 (2)	0.005 (3)	0.003 (2)
C17	0.113 (6)	0.085 (5)	0.065 (4)	0.032 (4)	0.044 (4)	0.006 (4)
C18	0.233 (11)	0.119 (7)	0.043 (4)	−0.021 (7)	0.003 (5)	−0.036 (4)
C19	0.035 (3)	0.054 (3)	0.095 (4)	−0.006 (2)	0.006 (3)	−0.039 (3)
C20	0.024 (2)	0.112 (5)	0.097 (5)	−0.004 (3)	0.004 (3)	−0.063 (4)
C21	0.058 (4)	0.140 (6)	0.051 (4)	0.030 (4)	0.016 (3)	−0.012 (4)
C22	0.048 (3)	0.077 (4)	0.049 (3)	0.000 (3)	0.004 (2)	0.012 (3)
C23	0.097 (5)	0.066 (4)	0.089 (5)	0.043 (4)	0.030 (4)	0.025 (4)
C24	0.049 (3)	0.047 (3)	0.168 (7)	−0.006 (3)	0.019 (4)	−0.049 (4)
Ag1	0.0601 (2)	0.03759 (19)	0.02085 (17)	−0.00057 (16)	−0.00066 (15)	−0.00184 (13)
Mo1	0.02685 (18)	0.02148 (17)	0.01861 (17)	−0.00241 (13)	−0.00162 (13)	0.00100 (13)
S1	0.0454 (6)	0.0238 (5)	0.0258 (5)	−0.0003 (5)	0.0007 (4)	0.0051 (4)
S2	0.0559 (7)	0.0213 (5)	0.0283 (5)	−0.0043 (5)	0.0002 (5)	0.0018 (4)
S3	0.0325 (6)	0.0505 (7)	0.0301 (6)	0.0137 (5)	−0.0057 (5)	−0.0035 (5)
S4	0.0312 (5)	0.0484 (6)	0.0297 (6)	−0.0064 (5)	0.0017 (4)	−0.0029 (5)

### Geometric parameters (Å, °)

**Table:**

Y1—O3	2.233 (3)	C6—H6A	0.9600
Y1—O2	2.242 (2)	C6—H6B	0.9600
Y1—O4	2.245 (3)	C6—H6C	0.9600
Y1—O1	2.263 (2)	C7—H7A	0.9600
Y1—O8	2.442 (3)	C7—H7B	0.9600
Y1—O9	2.465 (3)	C7—H7C	0.9600
Y1—O5	2.471 (3)	C8—H8A	0.9600
Y1—O6	2.472 (3)	C8—H8B	0.9600
Y1—N14	2.871 (3)	C8—H8C	0.9600
Y1—N13	2.888 (3)	C9—H9A	0.9600
P1—O1	1.482 (3)	C9—H9B	0.9600
P1—N2	1.628 (4)	C9—H9C	0.9600
P1—N1	1.642 (4)	C10—H10A	0.9600
P1—N3	1.656 (4)	C10—H10B	0.9600
P2—O2	1.492 (3)	C10—H10C	0.9600
P2—N6	1.620 (4)	C11—H11A	0.9600
P2—N5	1.638 (4)	C11—H11B	0.9600
P2—N4	1.639 (3)	C11—H11C	0.9600
P3—O3	1.479 (3)	C12—H12A	0.9600
P3—N9	1.609 (5)	C12—H12B	0.9600
P3—N8	1.616 (4)	C12—H12C	0.9600
P3—N7	1.652 (5)	C13—H13A	0.9600
P4—O4	1.498 (3)	C13—H13B	0.9600
P4—N12	1.619 (4)	C13—H13C	0.9600
P4—N10	1.622 (3)	C14—H14A	0.9600
P4—N11	1.644 (4)	C14—H14B	0.9600
O5—N13	1.271 (4)	C14—H14C	0.9600
O6—N13	1.270 (4)	C15—H15A	0.9600
O7—N13	1.219 (4)	C15—H15B	0.9600
O8—N14	1.272 (4)	C15—H15C	0.9600
O9—N14	1.268 (4)	C16—H16A	0.9600
O10—N14	1.227 (4)	C16—H16B	0.9600
N1—C1	1.444 (6)	C16—H16C	0.9600
N1—C2	1.475 (5)	C17—H17A	0.9600
N2—C3	1.456 (6)	C17—H17B	0.9600
N2—C4	1.475 (6)	C17—H17C	0.9600
N3—C5	1.439 (7)	C18—H18A	0.9600
N3—C6	1.456 (7)	C18—H18B	0.9600
N4—C7	1.461 (5)	C18—H18C	0.9600
N4—C8	1.465 (5)	C19—H19A	0.9600
N5—C10	1.456 (6)	C19—H19B	0.9600
N5—C9	1.472 (6)	C19—H19C	0.9600
N6—C11	1.447 (7)	C20—H20A	0.9600
N6—C12	1.476 (7)	C20—H20B	0.9600
N7—C13	1.422 (7)	C20—H20C	0.9600
N7—C14	1.461 (7)	C21—H21A	0.9600
N8—C15	1.466 (6)	C21—H21B	0.9600
N8—C16	1.480 (6)	C21—H21C	0.9600
N9—C17	1.427 (8)	C22—H22A	0.9600
N9—C18	1.466 (7)	C22—H22B	0.9600
N10—C19	1.440 (5)	C22—H22C	0.9600
N10—C20	1.474 (6)	C23—H23A	0.9600
N11—C22	1.469 (6)	C23—H23B	0.9600
N11—C21	1.470 (6)	C23—H23C	0.9600
N12—C23	1.446 (7)	C24—H24A	0.9600
N12—C24	1.468 (7)	C24—H24B	0.9600
C1—H1A	0.9600	C24—H24C	0.9600
C1—H1B	0.9600	Ag1—S2	2.4861 (11)
C1—H1C	0.9600	Ag1—S1	2.5576 (11)
C2—H2A	0.9600	Ag1—S4	2.6129 (13)
C2—H2B	0.9600	Ag1—S3	2.6193 (13)
C2—H2C	0.9600	Ag1—Mo1	2.9274 (6)
C3—H3A	0.9600	Ag1—Mo1^i^	2.9640 (7)
C3—H3B	0.9600	Mo1—S3^ii^	2.1899 (12)
C3—H3C	0.9600	Mo1—S4^ii^	2.2023 (13)
C4—H4A	0.9600	Mo1—S1	2.2036 (10)
C4—H4B	0.9600	Mo1—S2	2.2106 (11)
C4—H4C	0.9600	Mo1—Ag1^ii^	2.9640 (7)
C5—H5A	0.9600	S3—Mo1^i^	2.1899 (12)
C5—H5B	0.9600	S4—Mo1^i^	2.2023 (12)
C5—H5C	0.9600		
			
O3—Y1—O2	92.78 (10)	N3—C5—H5C	109.5
O3—Y1—O4	88.84 (10)	H5A—C5—H5C	109.5
O2—Y1—O4	157.00 (10)	H5B—C5—H5C	109.5
O3—Y1—O1	157.93 (10)	N3—C6—H6A	109.5
O2—Y1—O1	93.57 (9)	N3—C6—H6B	109.5
O4—Y1—O1	93.46 (10)	H6A—C6—H6B	109.5
O3—Y1—O8	128.62 (9)	N3—C6—H6C	109.5
O2—Y1—O8	79.89 (10)	H6A—C6—H6C	109.5
O4—Y1—O8	81.21 (10)	H6B—C6—H6C	109.5
O1—Y1—O8	73.36 (9)	N4—C7—H7A	109.5
O3—Y1—O9	76.50 (9)	N4—C7—H7B	109.5
O2—Y1—O9	77.69 (9)	H7A—C7—H7B	109.5
O4—Y1—O9	80.40 (9)	N4—C7—H7C	109.5
O1—Y1—O9	125.54 (9)	H7A—C7—H7C	109.5
O8—Y1—O9	52.18 (9)	H7B—C7—H7C	109.5
O3—Y1—O5	78.98 (9)	N4—C8—H8A	109.5
O2—Y1—O5	127.25 (9)	N4—C8—H8B	109.5
O4—Y1—O5	75.56 (9)	H8A—C8—H8B	109.5
O1—Y1—O5	80.35 (9)	N4—C8—H8C	109.5
O8—Y1—O5	143.55 (9)	H8A—C8—H8C	109.5
O9—Y1—O5	145.77 (9)	H8B—C8—H8C	109.5
O3—Y1—O6	79.78 (10)	N5—C9—H9A	109.5
O2—Y1—O6	75.51 (9)	N5—C9—H9B	109.5
O4—Y1—O6	127.24 (9)	H9A—C9—H9B	109.5
O1—Y1—O6	81.39 (9)	N5—C9—H9C	109.5
O8—Y1—O6	143.31 (9)	H9A—C9—H9C	109.5
O9—Y1—O6	142.99 (9)	H9B—C9—H9C	109.5
O5—Y1—O6	51.74 (9)	N5—C10—H10A	109.5
O3—Y1—N14	102.59 (10)	N5—C10—H10B	109.5
O2—Y1—N14	76.41 (10)	H10A—C10—H10B	109.5
O4—Y1—N14	80.85 (10)	N5—C10—H10C	109.5
O1—Y1—N14	99.45 (10)	H10A—C10—H10C	109.5
O8—Y1—N14	26.13 (9)	H10B—C10—H10C	109.5
O9—Y1—N14	26.09 (9)	N6—C11—H11A	109.5
O5—Y1—N14	156.34 (10)	N6—C11—H11B	109.5
O6—Y1—N14	151.90 (9)	H11A—C11—H11B	109.5
O3—Y1—N13	75.90 (10)	N6—C11—H11C	109.5
O2—Y1—N13	101.34 (10)	H11A—C11—H11C	109.5
O4—Y1—N13	101.30 (10)	H11B—C11—H11C	109.5
O1—Y1—N13	82.13 (9)	N6—C12—H12A	109.5
O8—Y1—N13	155.48 (9)	N6—C12—H12B	109.5
O9—Y1—N13	152.30 (9)	H12A—C12—H12B	109.5
O5—Y1—N13	25.97 (9)	N6—C12—H12C	109.5
O6—Y1—N13	25.95 (9)	H12A—C12—H12C	109.5
N14—Y1—N13	177.29 (10)	H12B—C12—H12C	109.5
O1—P1—N2	109.25 (18)	N7—C13—H13A	109.5
O1—P1—N1	108.25 (16)	N7—C13—H13B	109.5
N2—P1—N1	115.7 (2)	H13A—C13—H13B	109.5
O1—P1—N3	116.87 (18)	N7—C13—H13C	109.5
N2—P1—N3	103.3 (2)	H13A—C13—H13C	109.5
N1—P1—N3	103.61 (19)	H13B—C13—H13C	109.5
O2—P2—N6	110.91 (19)	N7—C14—H14A	109.5
O2—P2—N5	111.61 (17)	N7—C14—H14B	109.5
N6—P2—N5	106.7 (2)	H14A—C14—H14B	109.5
O2—P2—N4	108.68 (16)	N7—C14—H14C	109.5
N6—P2—N4	109.63 (19)	H14A—C14—H14C	109.5
N5—P2—N4	109.31 (19)	H14B—C14—H14C	109.5
O3—P3—N9	111.4 (2)	N8—C15—H15A	109.5
O3—P3—N8	109.84 (18)	N8—C15—H15B	109.5
N9—P3—N8	108.1 (2)	H15A—C15—H15B	109.5
O3—P3—N7	108.8 (2)	N8—C15—H15C	109.5
N9—P3—N7	109.0 (3)	H15A—C15—H15C	109.5
N8—P3—N7	109.6 (2)	H15B—C15—H15C	109.5
O4—P4—N12	119.37 (18)	N8—C16—H16A	109.5
O4—P4—N10	107.70 (16)	N8—C16—H16B	109.5
N12—P4—N10	104.7 (2)	H16A—C16—H16B	109.5
O4—P4—N11	107.76 (18)	N8—C16—H16C	109.5
N12—P4—N11	103.0 (2)	H16A—C16—H16C	109.5
N10—P4—N11	114.7 (2)	H16B—C16—H16C	109.5
P1—O1—Y1	161.37 (17)	N9—C17—H17A	109.5
P2—O2—Y1	168.21 (17)	N9—C17—H17B	109.5
P3—O3—Y1	167.49 (17)	H17A—C17—H17B	109.5
P4—O4—Y1	158.56 (16)	N9—C17—H17C	109.5
N13—O5—Y1	95.7 (2)	H17A—C17—H17C	109.5
N13—O6—Y1	95.6 (2)	H17B—C17—H17C	109.5
N14—O8—Y1	96.1 (2)	N9—C18—H18A	109.5
N14—O9—Y1	95.1 (2)	N9—C18—H18B	109.5
C1—N1—C2	113.2 (4)	H18A—C18—H18B	109.5
C1—N1—P1	120.4 (3)	N9—C18—H18C	109.5
C2—N1—P1	120.7 (3)	H18A—C18—H18C	109.5
C3—N2—C4	115.1 (4)	H18B—C18—H18C	109.5
C3—N2—P1	119.9 (3)	N10—C19—H19A	109.5
C4—N2—P1	120.6 (3)	N10—C19—H19B	109.5
C5—N3—C6	110.9 (5)	H19A—C19—H19B	109.5
C5—N3—P1	123.4 (4)	N10—C19—H19C	109.5
C6—N3—P1	121.1 (4)	H19A—C19—H19C	109.5
C7—N4—C8	114.6 (3)	H19B—C19—H19C	109.5
C7—N4—P2	119.8 (3)	N10—C20—H20A	109.5
C8—N4—P2	122.3 (3)	N10—C20—H20B	109.5
C10—N5—C9	114.8 (4)	H20A—C20—H20B	109.5
C10—N5—P2	125.4 (3)	N10—C20—H20C	109.5
C9—N5—P2	118.8 (3)	H20A—C20—H20C	109.5
C11—N6—C12	115.4 (5)	H20B—C20—H20C	109.5
C11—N6—P2	119.3 (4)	N11—C21—H21A	109.5
C12—N6—P2	124.3 (4)	N11—C21—H21B	109.5
C13—N7—C14	113.5 (5)	H21A—C21—H21B	109.5
C13—N7—P3	121.7 (4)	N11—C21—H21C	109.5
C14—N7—P3	123.7 (5)	H21A—C21—H21C	109.5
C15—N8—C16	115.1 (4)	H21B—C21—H21C	109.5
C15—N8—P3	124.1 (4)	N11—C22—H22A	109.5
C16—N8—P3	118.7 (3)	N11—C22—H22B	109.5
C17—N9—C18	117.3 (6)	H22A—C22—H22B	109.5
C17—N9—P3	119.7 (4)	N11—C22—H22C	109.5
C18—N9—P3	123.0 (6)	H22A—C22—H22C	109.5
C19—N10—C20	113.6 (4)	H22B—C22—H22C	109.5
C19—N10—P4	122.1 (3)	N12—C23—H23A	109.5
C20—N10—P4	121.9 (3)	N12—C23—H23B	109.5
C22—N11—C21	111.9 (4)	H23A—C23—H23B	109.5
C22—N11—P4	118.7 (3)	N12—C23—H23C	109.5
C21—N11—P4	120.8 (3)	H23A—C23—H23C	109.5
C23—N12—C24	112.6 (5)	H23B—C23—H23C	109.5
C23—N12—P4	121.5 (4)	N12—C24—H24A	109.5
C24—N12—P4	123.2 (4)	N12—C24—H24B	109.5
O7—N13—O6	121.5 (3)	H24A—C24—H24B	109.5
O7—N13—O5	122.3 (3)	N12—C24—H24C	109.5
O6—N13—O5	116.1 (3)	H24A—C24—H24C	109.5
O7—N13—Y1	172.4 (3)	H24B—C24—H24C	109.5
O6—N13—Y1	58.41 (18)	S2—Ag1—S1	94.11 (3)
O5—N13—Y1	58.34 (17)	S2—Ag1—S4	120.83 (4)
O10—N14—O9	122.2 (3)	S1—Ag1—S4	119.99 (4)
O10—N14—O8	121.3 (4)	S2—Ag1—S3	120.28 (4)
O9—N14—O8	116.4 (3)	S1—Ag1—S3	117.40 (4)
O10—N14—Y1	175.3 (3)	S4—Ag1—S3	86.92 (4)
O9—N14—Y1	58.80 (18)	S2—Ag1—Mo1	47.34 (3)
O8—N14—Y1	57.76 (18)	S1—Ag1—Mo1	46.78 (2)
N1—C1—H1A	109.5	S4—Ag1—Mo1	137.27 (3)
N1—C1—H1B	109.5	S3—Ag1—Mo1	135.75 (3)
H1A—C1—H1B	109.5	S2—Ag1—Mo1^i^	150.72 (3)
N1—C1—H1C	109.5	S1—Ag1—Mo1^i^	115.14 (3)
H1A—C1—H1C	109.5	S4—Ag1—Mo1^i^	45.99 (3)
H1B—C1—H1C	109.5	S3—Ag1—Mo1^i^	45.67 (3)
N1—C2—H2A	109.5	Mo1—Ag1—Mo1^i^	161.916 (13)
N1—C2—H2B	109.5	S3^ii^—Mo1—S4^ii^	110.05 (5)
H2A—C2—H2B	109.5	S3^ii^—Mo1—S1	108.06 (5)
N1—C2—H2C	109.5	S4^ii^—Mo1—S1	108.67 (5)
H2A—C2—H2C	109.5	S3^ii^—Mo1—S2	108.03 (5)
H2B—C2—H2C	109.5	S4^ii^—Mo1—S2	108.48 (4)
N2—C3—H3A	109.5	S1—Mo1—S2	113.54 (4)
N2—C3—H3B	109.5	S3^ii^—Mo1—Ag1	125.42 (4)
H3A—C3—H3B	109.5	S4^ii^—Mo1—Ag1	124.52 (4)
N2—C3—H3C	109.5	S1—Mo1—Ag1	57.75 (3)
H3A—C3—H3C	109.5	S2—Mo1—Ag1	55.79 (3)
H3B—C3—H3C	109.5	S3^ii^—Mo1—Ag1^ii^	58.82 (4)
N2—C4—H4A	109.5	S4^ii^—Mo1—Ag1^ii^	58.57 (4)
N2—C4—H4B	109.5	S1—Mo1—Ag1^ii^	148.33 (3)
H4A—C4—H4B	109.5	S2—Mo1—Ag1^ii^	98.13 (3)
N2—C4—H4C	109.5	Ag1—Mo1—Ag1^ii^	153.915 (13)
H4A—C4—H4C	109.5	Mo1—S1—Ag1	75.47 (3)
H4B—C4—H4C	109.5	Mo1—S2—Ag1	76.87 (3)
N3—C5—H5A	109.5	Mo1^i^—S3—Ag1	75.50 (4)
N3—C5—H5B	109.5	Mo1^i^—S4—Ag1	75.45 (4)
H5A—C5—H5B	109.5		

Symmetry codes: (i) *x*, −*y*+1/2, *z*−1/2; (ii) *x*, −*y*+1/2, *z*+1/2.

### Hydrogen-bond geometry (Å, °)

**Table:**

*D*—H···*A*	*D*—H	H···*A*	*D*···*A*	*D*—H···*A*
C5—H5A···S2^iii^	0.96	2.79	3.710 (6)	160
C16—H16A···O10^i^	0.96	2.49	3.292 (6)	141
C18—H18A···O10^iv^	0.96	2.54	3.470 (9)	162

Symmetry codes: (iii) −*x*+1, −*y*, −*z*+1; (i) *x*, −*y*+1/2, *z*−1/2; (iv) *x*, *y*, *z*−1.
